# Overall adjustment acupuncture for postmenopausal osteoporosis (PMOP): a study protocol for a randomized sham-controlled trial

**DOI:** 10.1186/s13063-020-04435-7

**Published:** 2020-06-03

**Authors:** Z. Q. Ren, Y. F. Wang, G. F. Ao, H. X. Chen, M. Huang, M. X. Lai, H. D. Zhao, R. Zhao

**Affiliations:** 1 Nanjing University of Chinese Medicine, No.138 Xianlin Road, Nanjing, 210046 China; 2 The First Affiliated Hospital of Dali University, No. 32 Jiashibo Road, Dali, 671000 Yunnan Province China; 3 School of Acupuncture-Tuina and Rehabilitation, Yunnan University of Chinese Medicine, No.1076 Yuhua Road, Chenggong District, Kunming, 650500 Yunnan Province China; 4 Department of Acupuncture, Kunming Municipal Hospital of Traditional Chinese Medicine, 25 Dongfeng Road, Panlong District, Kunming, 650011 Yunnan Province China; 5 The First Affiliated Hospital of Yunnan University of Chinese Medicine, No.120 Guanghua Road, Wuhua District, Kunming, 650032 Yunnan Province China

**Keywords:** Osteoporosis, PMOP, Acupuncture, Sham acupuncture, Protocol, Randomised controlled trial

## Abstract

**Background:**

Osteoporosis is becoming more prevalent in aging societies worldwide, and the economic burden attributable to osteoporotic fractures is substantial. The medications presently available to treat osteoporosis have side effects. Acupuncture is widely used for treating osteoporotic postmenopausal women because it is non-invasive and has fewer side effects, but the powerful clinical evidence for its efficacy remains insufficient. Our study intends to explore the effect of overall adjustment acupuncture (OA) in the treatment of postmenopausal osteoporosis (PMOP).

**Methods/design:**

This study is a randomized, sham-controlled, patient- and assessor-blinded trial and aims to evaluate the effect of OA in women with PMOP. We will recruit 104 women aged 45–70 years with a diagnosis of PMOP. Participants will be randomly allocated in a 1:1 ratio to the OA group and the sham acupuncture (SA) group. Both groups will receive real herbal medicine treatment as a basic treatment twice a day for 3 months, the OA group receives real acupuncture treatment and the SA group receives placebo acupuncture treatment (non-penetrating, sham skin-needle therapy, sham cupping). All patients will receive acupuncture treatment twice per week for 3 months. The primary outcome is bone mineral density (BMD) and the secondary outcomes include estradiol (E2), follicle-stimulating hormone (FSH), bone gla protein (BGP), bone alkaline phosphatase (BALP), total antioxidant capacity (TAC), advanced oxidation protein products (AOPP), PPARγ, β-catenin, FoxO3a levels, visual analog pain scale score (VAS), Traditional Chinese medicine (TCM) syndrome scores and quality of daily life score (QOL). Outcome measures will be collected at baseline, middle of the treatment (1.5 months), the end of treatment (3 months). The present protocol followed the SPIRIT guidelines and fulfills the SPIRIT Checklist.

**Conclusion:**

This study will be conducted to compare the efficacy of OA versus SA. This trial should help to evaluate whether OA can effectively prevent and treat PMOP by improving the estrogen levels of postmenopausal women. The mechanism is to improve the imbalance of osteogenic differentiation and lipogenesis of bone-marrow cells under oxidative stress.

**Trial registration:**

Chinese Clinical Trial Registry, ID: ChiCTR1800017581. Registered on 5 August 2018. URL: http://www.chictr.org.cn.

## Introduction

The high incidence and the associated pain and fractures of postmenopausal osteoporosis (PMOP) have seriously threatened many women’s physical and mental health, as well as their life quality. According to statistics, about one quarter of perimenopausal women will develop osteoporosis, and these women will lose bone mass rapidly within 5 years post menopause; the premenopausal loss is two to three times that of younger women [1].^.^The prevention and treatment of PMOP is directly related to the physical and mental health outcomes of most postmenopausal women, and this has great social and practical significance.

Postmenopausal osteoporosis is caused by a decline of ovarian function, rapid aging of the body, decreased estrogen levels, hyperabsorptive function, and compensatory enhancement of osteoblastic bone formation. All of these factors result in a high degree of bone metabolism. The ensuing state of negative balance eventually leads to a decrease in bone mass, destruction of bone microstructure, reduction of bone strength, and a type of systemic bone metabolic disease that is prone to fractures [2]. In recent years, it has been recognized that PMOP is caused by aging, and estrogen deficiency. As the basic pathogenesis of aging, oxidative stress plays an important role in the occurrence of related diseases. Estrogen is a powerful antioxidant, and increased accumulation of reactive oxygen species (ROS) induces oxidative stress, eventually lead to PMOP [3]. It is generally believed that with the increase of ROS and oxidative damage, the formation and survival of bone osteoblasts decreases, while osteoclastic differentiation and activity increase [4, 5]. Some researches show that acupuncture which is performed on the basis that conventional orally administered calcium improves bone density and serum estradiol levels in PMOP patients. The mechanism may be that acupuncture can increase estrogen levels and promote osseous calcium deposition, can affect the bone turnover in order to increase bone density [6]. Oxidative stress-mediated balance of the FoxO3a-β-catenin-PPARγ signal axis may play an important role in bone-marrow mesenchymal stem cells (BMSCs). β-catenin plays a central role in the regulation of osteogenic differentiation and osteoblast activity in the bone-marrow-fat environment [7].

At present, the prevention and treatment of osteoporosis (POP) mainly depends on pharmaceuticals, but there are certain side effects [8], and their long-term effect is unsatisfactory. Estrogen therapy, as a complementary replacement therapy for PMOP, has a long-term application with definite effects, but may often cause adverse events such as breast cancer or endometrial cancer [9]. The side effects of other medicines which are commonly used in clinical practice have also become increasingly prominent. Adverse reactions, such as atypical femoral fractures and mandibular necrosis, caused by the use of bipionate have also attracted increasing attention [10]. The findings have affected their acceptance by many patients. Therefore, it is significant to find a safe, effective, ecologically green, natural, and non-side-effect-causing treatment. Also, first-line clinical medicine is mostly focused on inhibiting the abnormally active bone resorption dominated by osteoclasts. Simply inhibiting osteolysis is difficult to fundamentally treat osteoporosis. The mature growth of osteoblasts is the basis of bone health, so we should pay more attention to the research on methods of promoting bone formation.

Acupuncture therapy is a important therapy for the prevention and treatment of chronic metabolic diseases in traditional Chinese medicine (TCM). The clinical treatment of acupuncture for this disease is mainly to strengthen the kidney and bones, while regulating the spleen and kidney. The clinical effect of acupuncture on this disease is definite, and it can significantly improve the symptoms of low-back pain, limb weakness and kidney deficiency caused by PMOP. Acupuncture can not only alleviate and improve clinical symptoms, such as pain, in patients with osteoporosis, but also can prevent osteoporosis by regulating the levels of endocrine hormones in the body, increasing bone density, and improving abnormal bone metabolism [11, 12].

The currently recognized principles of TCM treatment of osteoporosis are syndrome differentiation, overall regulation, prevention and treatment [13]. “Overall adjustment acupuncture” (OA) is a kind of acupuncture treatment based on TCM, which is an effective, systematic, comprehensive and multi-pathway treatment of osteoporosis. The OA is based on more than 20 years of clinical experience and experimental verification. It combines the main etiology and pathogenesis of osteoporosis with kidney and spleen deficiency. It involves a systematic process for osteoporosis and it combines acupuncture, moxibustion, and skin-needling organically to stimulate the meridian system such as those of the skin, collaterals, and meridians. According to the full regulation of this therapy, osteoporosis has been treated by improving clinical symptoms and patients’ quality of life through multiple channels [14]. During the early stages of clinical treatment, it was found that the OA method has a good effect on PMOP, but a systematic and comprehensive clinical study has not been conducted and, thus, its mechanism of action is not clear. This trial will study the efficacy, safety and mechanism of acupuncture therapy on PMOP. There are few reports on the treatment of PMOP by sham acupuncture (SA) as a control group. Our trial hypothesis is that overall regulation acupuncture treats PMOP by increasing the estrogen levels of postmenopausal women and improving the imbalance of osteogenic and adipogenic differentiation of BMSCs caused by oxidative stress in the human body.

## Methods/design

### Study design

**Fig. 1:**
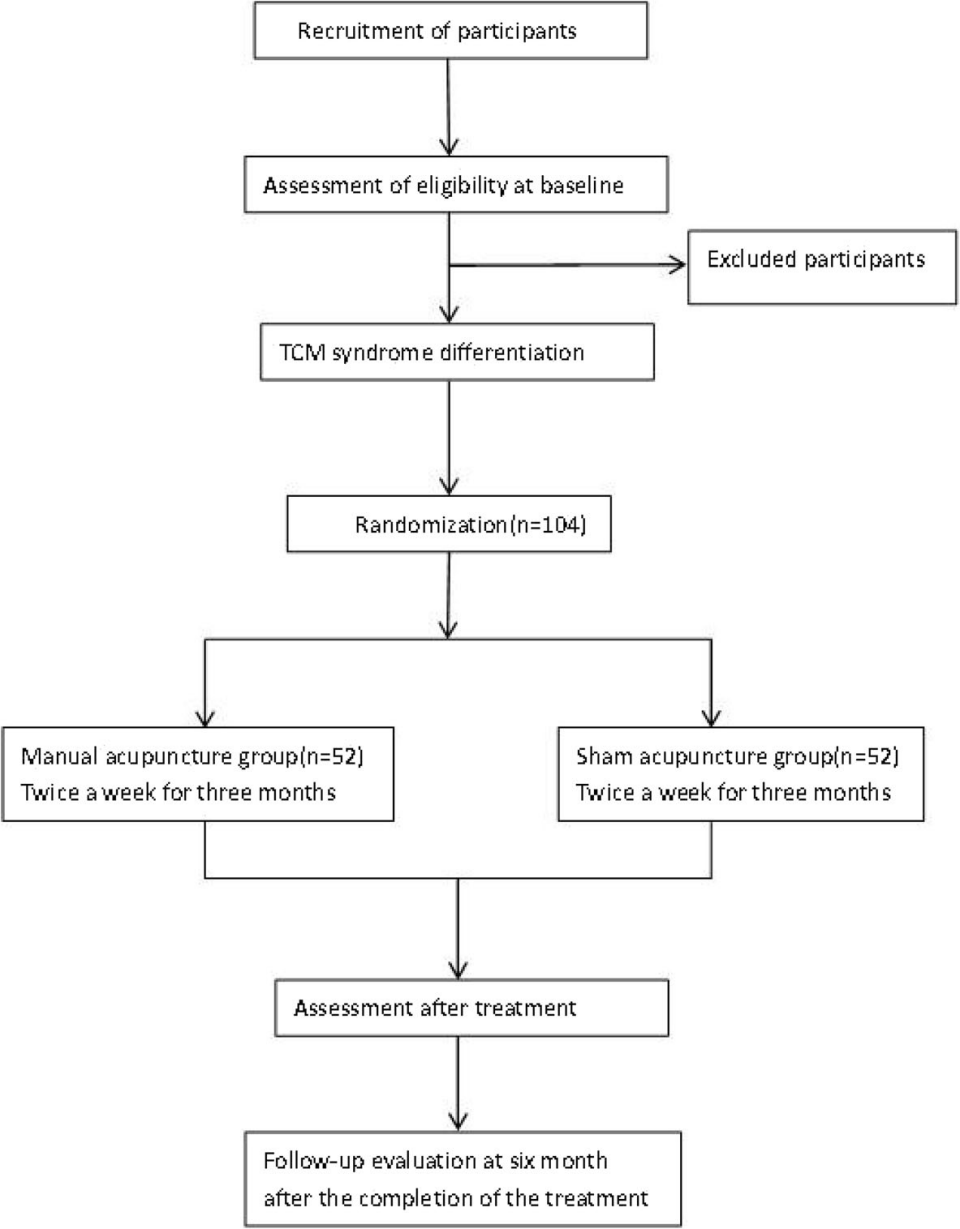
Study flow chart

This is a randomized, sham-controlled, patient- and assessor-blinded trial. A target sample of 104 participants will be recruited from the Acupuncture Clinic at the First Affiliated Hospital of Dali University and Yunnan Provincial Hospital of Traditional Chinese Medicine. The present protocol follows the Standard Protocol Items: Recommendations for Interventional Trials (SPIRIT) guidelines and fulfills the SPIRIT Checklist (Additional file 1). The trial flow chart is shown in Fig. 1. The protocol is in line with the principles of the Declaration of Helsinki and has been approved by Institution Review Board (IRB) of Yunnan Provincial Hospital and Traditional Chinese Medicine (approval no. 2018–003-01). This trial was registered at the Chinese Clinical Trial Registry (ChiCTR1800017581). Any changes which need to be made in the trial protocol will be communicated to all researchers, the Ethics Committees, and the trial registry. Written informed consent will be signed by each participant.

Participants who voluntarily sign the informed consent, and who are eligible for this study, will be assigned randomly to one of two groups (the acupuncture group or the SA group) in a 1:1 allocation. On the consent form, participants will be asked if they agree to the use of their data should they choose to withdraw from the trial. Participants will also be asked for permission for the research team to share any relevant data with people from the Dali University who are taking part in the research or from regulatory authorities, where relevant. The study participants were recruited competitively from two sites of the Acupuncture and Moxibustion Department of Yunnan Province Hospital of TCM and the First Affiliated Hospital of Dali University. A total of 104 postmenopausal women with osteoporosis were recruited via broadcast media, newspapers, posters, and the Internet homepages of the participating hospitals, as well as via advertisements placed in nearby welfare centers.

### Randomization and allocation concealment

For randomization, an independent, blinded statistician will generate random numbers using the SAS randomization program (version 9.4; SAS Institute, Inc., Cary, NC, USA). The manufacturer will supply the hospital with investigational treatments in consecutively numbered drug containers with identical packaging to conceal treatment allocation. Blinded researchers in each hospital will enroll and assign participants to one of the two study groups.

### Sample size and blinding

According to the preliminary research [15], we hypothesize that the effective rate after OA is 77.8% and the effective rate after SA is 50%. The equation that we used for this was *α* = 0.05, *β* = 0.20 and we determined that a sample size of 42 patients in each group would be sufficient to detect the statistical difference between the two groups, allowing for a 20% withdrawal rate. We will plan to enroll a total of 104 participants with 52 participants in each group. According to the random number sequence generated by computer, the patients will be randomly divided into either the OA group or the SA group in a 1:1 ratio.

The participants, outcome assessors, study monitors, data managers, and statisticians will be blinded to treatment allocation. If possible, the study participants will be advised to avoid discussing the investigational treatments with other participants. Blinding will be maintained until the 104 participants have completed the study. The success of double-blinding will be assessed at the final visit. Due to the special nature of acupuncture, acupuncturists cannot be blinded to the treatment. The database will then be locked. An emergency code for each individual has been provided to the study researchers and will be disclosed only if it must be known whether a patient is receiving acupuncture treatment or SA in order to manage a serious adverse event.

### Inclusion criteria

Participants who meet all the following requirements will be allowed to enrol

1. Women aged 45–70 years
2. Patients who meet the diagnostic criteria for PMOP
  - Pain occurs mostly with the hips, lower back and ribs, and is of slow onset
  - A brittle fracture occurs with only slight external force
  - There is an obvious tender point on the lower back or there is a large area of tenderness
  - Scoliosis, kyphosis and other deformities exist
  - DXA (dual-energy x-ray absorptiometry) is used to detect a femoral-neck bone-mineral density lower than − 2.5. Severe osteoporosis is accompanied by two or more fractures
  - Imaging examination showed obvious osteoporosis or a fragility fracture
  - Non-secondary osteoporosis is present
3. Patients who meet the “atrophic debility of bones” in the Chinese medicine diagnostic criteria
4. Natural menopause for more than 1 year
5. Other medications or other related interventions for PMOP were not received within 4–6 months
6. Patients voluntarily provided informed consent, and willingly signed the informed consent

### Exclusion criteria

Participants meeting any of the following criteria will be excluded:

1. Except for women with abnormal lumbar anatomy (such as severe scoliosis)
Exclude related endocrine disorders (such as diabetes, hyperthyroidism, hypothyroidism, thyroid cysts, etc.) that can cause secondary osteoporosis
After uterine or ovarian ablation surgery
Concomitant with diseases such as rickets, rheumatoid arthritis or other diseases that affect the dynamic balance of bone metabolism
In the past 3 months, the participant has taken drugs that can interfere with bone metabolism (such as glucocorticoids, calcitonin, estrogens, etc.);
Female patients with severe underlying diseases and mental illness who are unable to cooperate with treatment research programs
Drug or alcohol dependency/misuse
Those who had received acupuncture treatment
Those participating in other clinical trials

### Interventions

Both the OA and SA groups will receive acupuncture sessions for a total of 3 months. All the patients will receive herbal medicine twice a day for 3 months. The same physician (the Professor of Acupuncture in Yunnan Provincial Hospital and Traditional Chinese Medicine, who has 20 years of work experience) will slightly adjust the formula each week depending on the changes of the symptoms, pulse, and tongue coating of the patients (main herbal formula components are shown in Table 1). During the trial, patients will be classified by pattern differentiation: (1) spleen-kidney *Yang* deficiency will be given Yougui Pill; (2) liver-kidney *Yin* deficiency will be given Zuogui Pill; and (3) kidney deficiency and blood stasis will be given Bushen Huoxue decoction. Participants will be given all the herbs from the same hospital and the physician will instruct them how to decoct.

**Table 1:** Main herbal formula selection

Pattern	Formula	Composition
Spleen-kidney *Yang* deficiency	Yougui Pill	radix rehmanniae preparata, cortex cinnamomi, cornu cervi degelatinatum, rhizoma dioscoreae, fructus corni, fructus lycii, radix angelicae sinensis, cortex eucommiae, semen cuscutae, radix morindae officinalis, rhizoma drynaria, rhizoma sparganii
Spleen-kidney *Yin* deficiency	Zuogui Pill	radix rehmanniae preparata, rhizoma dioscoreae, fructus lycii, fructus corni, radix achyranthis bidentatae, deerhorn glue, deerhorn glue, radix morindae officinalis
Kidney deficiency and blood stasis	Bushen Huoxue decoction	radix rehmanniae preparata, semen cuscutae, cortex eucommiae, fructus lycii, radix angelicae sinensis, fructus corni, cistanches herba, commiphora myrrha, radix angelicae biseratae, radix angelicae sinensis

**Fig. 2:**
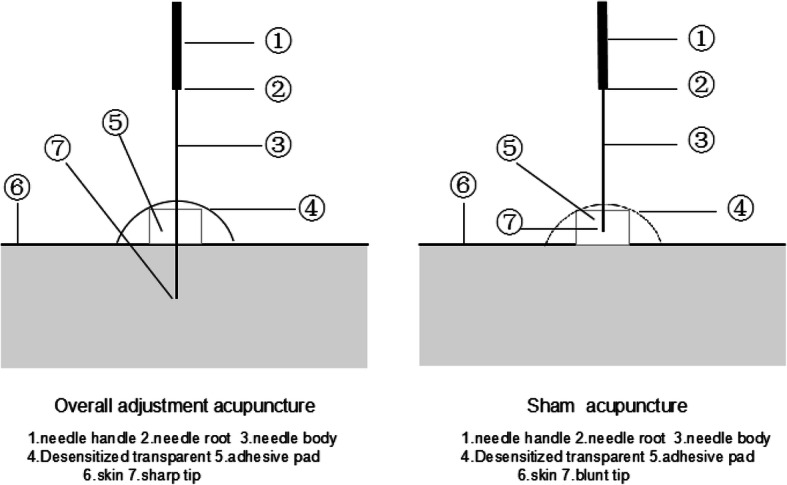
Schematic diagram of the acupuncture device

According to the OA and moxibustion [15], and taking opinions from Chinese acupuncture experts, the essential acupuncture points selected are as follows: BL11 (Dazhu), BL23 (Shenshu) and ST36 (Zusanli). The additional individualized acupuncture points will be chosen by the practitioners according to the patterns of identification: (1) kidney *Yang* deficiency will have added DU04 (Mingmen), GB34 (Guanyuan); (2) spleen-kidney *Yang* deficiency will have added DU04 (Mingmen), GB34 (Guanyuan); (2) liver-kidney *Yin* deficiency will have added SP06 (Sanyinjiao), LU11 (Taixi); (3) kidney deficiency and blood stasis will have added SP06 (Sanyinjiao), BL17 (Geshu) (Table 2). A placebo device [16] will be applied in both groups for better implementation of blinding. The schematic diagram of the acupuncture treatment is shown in Fig. 2.The acupuncture treatment in both groups will take 30 min per session, twice per week. Three months is the duration of a course of treatment, for a total of one course .

**Table 2:** Acupuncture point selection

Point	Location
BL11 (Dazhu)	In the spinal region, under the spinous process of the 1st thoracic vertebra, 1.5 cun lateral to the posterior midline
BL23 (Shenshu)	1.5 cun beside the spinous process of the 2nd lumbar vertebra
ST36 (Zusanli)	At the anterior aspect of the leg 3 cun inferior to ST35 (Dubi) on the line connecting ST35 (Dubi) to ST41 (Jiexi)
DU04 (Mingmen)	Between the spinous processes of the 2nd and 3rd lumbar vertebrae
GB34 (Guanyuan)	On the lower abdomen at the anterior midline, 3 cun below the umbilicus
SP06 (Sanyinjiao)	On the tibial aspect of the leg posterior to the medial border of the tibia, 3 cun superior to the prominence of the medial malleolus
LU11 (Taixi)	On the posteromedial aspect of the ankle in the depression between the prominence of the medial malleolus and the calcaneal tendon
BL17 (Geshu)	Under the spinous process of the 7th thoracic spine on the back, 1.5 cun lateral to the midline

### Overall adjustment acupuncture (OA) group

Acupuncture manipulation will be applied to the acupoints of the body until the patient achieves *Qi*, including soreness, soreness and distension, pain, numbness, tingling and even comfort. Huatuo brand needles (0.25 mm × 40 mm) are manufactured by the Suzhou Medical Device Company in Suzhou, Jiangsu Province, China. After the patient achieves *Qi* in Shenshu and Zusanli, warm acupuncture will be used, the 2 cm-long moxa-stick will be fixed on the handle of the needle and lit at the root of the needle and allowed to burn for 20 min.

The parameters of the skin-needle are set as follows: *Qixing* needle; tapping along the first side line of the urinary bladder meridian in the back; moderate stimulation will be applied for blood stasis and *Qi* stagnation type, until redness of the skin and petechiae are observed in the tapping area; mild stimulation is used for the other syndromes until redness in the skin is observed.

The parameters of the cupping are set as follows: moving cupping; smear the tapping spot evenly with Vaseline and move the cup along the first lateral line of the urinary bladder meridian, back and forth until red-blood stasis appears on the skin, according to the patient’s tolerance (feeling comfortable, without obvious pain). Retaining cupping: leave the cup for 8 to 10 min in Dazhui, Shenshu, and a severely painful area.

### Sham acupuncture (SA)group

**Fig. 3:**
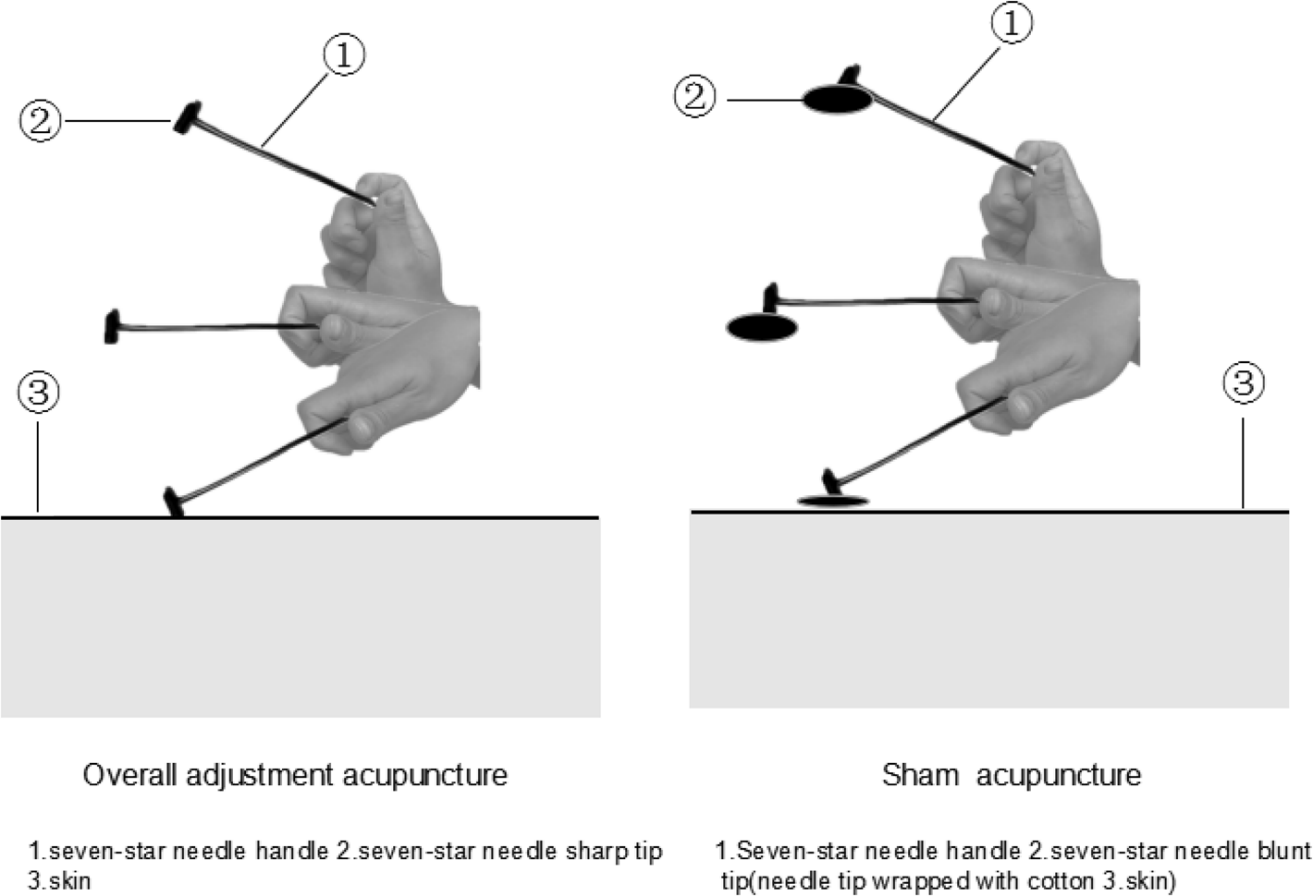
Schematic diagram of a skin needle

**Fig. 4:**
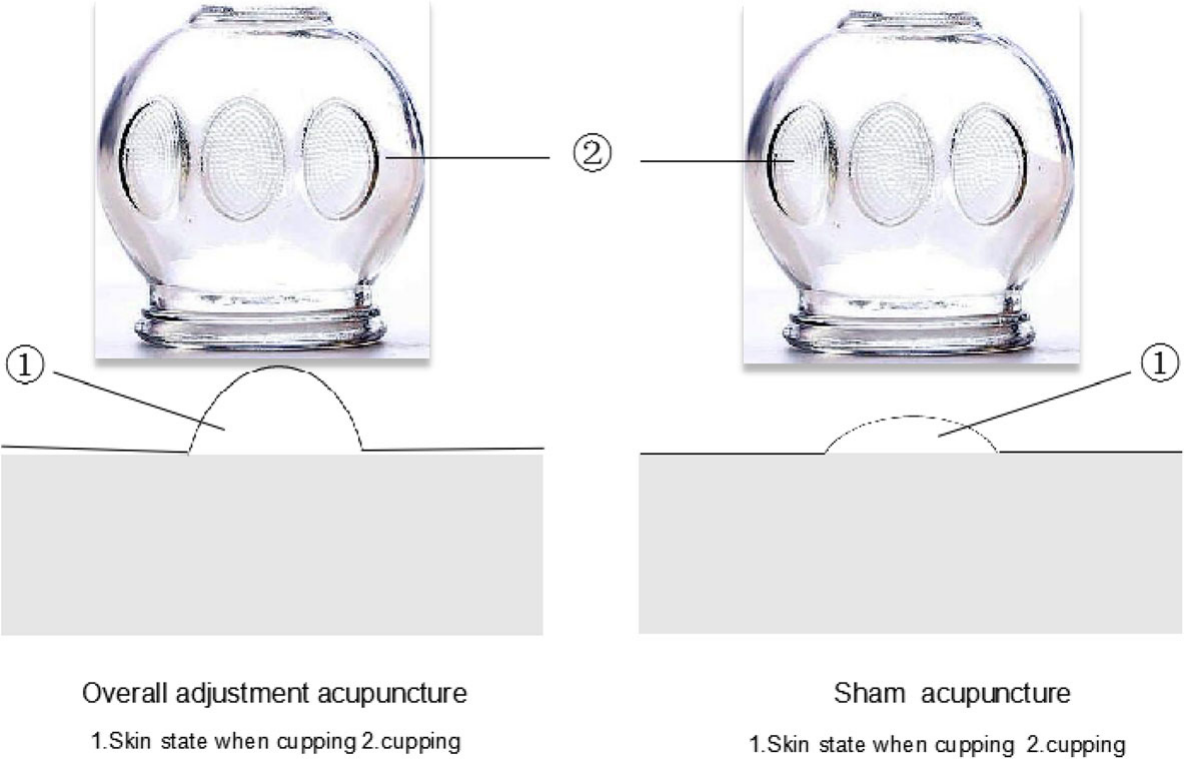
Schematic diagram of cupping

The procedure and duration of treatment in the SA group will be identical to the OA group except for the needles (0.25 × 25 mm with a blunt tip, manufactured by Suzhou Medical Device Company in Suzhou, Jiangsu Province, China). The *Qixing* needle tips are wrapped in cotton (shown in Fig. 3) are blunt-tipped and there will be no skin penetration or manual stimulation, but at the same time cupping is applied to the skin with a small negative pressure compared to that in the OA group (show in Fig. 4).

#### Practitioner background

The OA and SA treatments will be handled by acupuncturists who are registered and possess the TCM practitioner qualification certificate and who are specialized in acupuncture with more than 3 years of clinic experience. They will have studied acupuncture for more than 10 years and graduated from a TCM university. All operators will be trained in the overall adjustment of the needling procedure and will be advanced qualified, and will undergo intensive and customized training for a full understanding of the SA procedure and for using a sham needle device. The techniques for the entire treatment procedure will be standardized between practitioners.

### Concomitant and forbidden treatments

Drugs that have been taken for an indication other than osteoporosis for more than 30 days before the screening visit may be continued during the study period. Continuation of the same doses of health supplements, such as calcium or vitamin D, are permitted in this trial only if they have been taken continuously in the 3 months before the screening visit. Prohibited drugs include agents that can affect bone mineral density (BMD), such as other medications for osteoporosis, steroids, carbamazepine, phenytoin, phenobarbital, heparin, warfarin, thyroid hormones, gonadotropin-releasing hormone agonists, depot-medroxyprogesterone acetate, anticancer drugs, cyclosporine A, antidepressants, aluminum-containing antacids, aromatase inhibitors, antituberculosis drugs, thiazolidinedione, proton-pump inhibitors, antiretroviral drugs, and thiazide diuretics. Participants who have taken or are still taking any of these medications will be withdrawn from the study.

### Outcome measures

Outcomes will be collected at baseline, the middle of treatment, after treatment, and follow-up 6 months after the completion of the treatment. The overview of the outcome measurement at the different time points is shown in Table 3.

**Table 3:** Overview of study visits

		Study period						
		Enrollment	Allocation	Post allocation			Close out	Follow-up
	Time point (month)	− 1	0	1	2	3		6
Preparation	Enrollment							
	Informed consent	√						
	Eligibility screen	√						
	Allocation		√					
Intervention	OA			√	√	√		
	SA			√	√	√		
Assessment	Liver function Kidney function	√						
	T; BP; P; R	√		√	√	√		
	BMD	√						√
	E2; FSH	√						
	BGP; BALP	√						
	TAC; AOPP	√						
	FoxO3a; β-catenin PPARγ	√						
	VAS	√		√	√	√		√
	TCM syndrome	√			√			
	QOL	√			√			
	Adverse events						√	
	Data analysis						√	
	Fracture rate							√

*T* body temperature, *BP* blood pressure, *P* pulse rate, *R* respiration rate, *OA*, *SA*, *QOL* quality of life, *TCM* traditional Chinese Medicine, *VAS* visual analog scale, *PPAR*, *E2*, *TAC*, *AOPP*, *BGP*, *BALP*, *BMD*, *FSH*

### Primary outcome

The primary outcome is BMD. BMD is measured using dual-energy x-ray (DEXA) absorptiometry (DMS, Paris, France; model: CHALLENGER C 313). A BMD measurement of the lumbar vertebrae (L1–L4) in the two groups will be measured before and after treatment.

### Secondary outcomes

#### Pain score

The pain score will be assessed for the lower back. A visual analogue scale (VAS) will be used to measure each group before and after treatment (see attached Table 4).

**Table 4:**
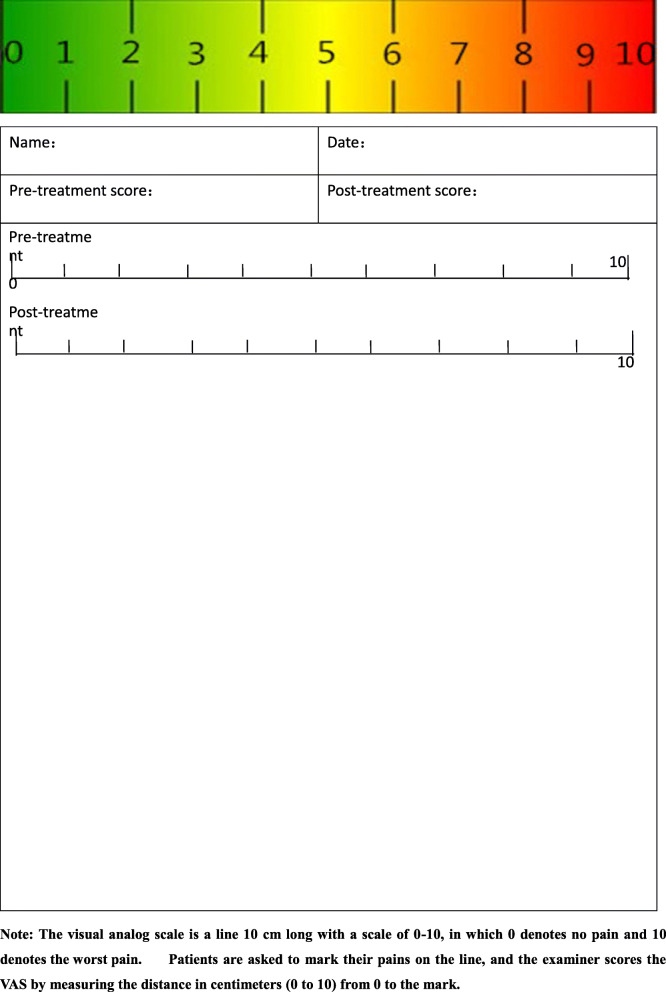
Visual analog scale (VAS)

Note: the visual analog scale is a line 10 cm long with a scale of 0–10, in which 0 denotes no pain and 10 denotes the worst pain. Patients are asked to mark their pains on the line, and the examiner scores the VAS by measuring the distance in centimeters (0 to 10) from 0 to the mark

The TCM syndrome scores will be recorded by the same professor once a week to adjust the acupuncture and herbal treatment [17] and evaluate the improvement of the patients’ TCM syndromes (see attached Table 5).

**Table 5:** Osteoporosis symptom grading scale (points)

Symptoms	None (0 points)	Mild (2 points)	Moderate (4 points)	Severe (6 points)
**Low-back pain**	**None**	**1–3°**	**4–6°**	**7–9°**
**Soreness and wea kness of waist and knees**	**None**	**After overwalking, I feel a little sore in my waist and knees**	**Somewhere in between**	**Soreness and wea kness of waist and knees continue to occur, and I do not want to stand andwalk**
**Lower limb pain**	**None**	**1–3°**	**4–6°**	**7–9°**
**Impotence and weakness in the lower limbs**	**None**	**After walking(≥ 1 km) I may have sore lower limbsoccasionally**	**After walking** **(300 m–1 km) I may feel weak and sore in my lower limbs**	**After standing and walking (< 300 m) My legs may immediately feel tired**
**Difficulty in walking**	**None**	**It is inconvenient for me to walk occasionally, and I do not feel discomfort walking less than 100 m**	**It is difficult for me to walk even for a short distance (10–100 m)**	**It is difficult for me to stand and walk. And I cannot walk for more than 10 m**
**Dizziness**	**None**	**I get dizzy occasionally, but dizziness does not affect my daily life**	**I get dizzy sometimes, and dizziness would get worse when I am tired, which affects my daily life**	**I get dizzy when I move, and sometimes even fall down, which seriously affects my daily life**
**Pre-treatment score**				
**Post-treatment score**				

**Note: osteoporosis symptom grading scale is a measure of pain intensity, using none, mild, moderate, and severe categories with specific scores. Specifically, no pain (0 points); mild pain, without affecting one’s work or life (2 points); moderate pain, affecting one’s work but not life (4 points); severe pain, affecting one’s work and life (6 points)**

#### Indicators for quality of life

Based on quality of life (QOL) and the 36-Item Short Form Survey (SF-36), combined with the specificity of osteoporosis and clinical situations, a self-rating scale in line with Chinese ethnicity was developed (see attached Table 6).

**Table 6:** Quality of life questionnaire for patients with osteoporosis

Items	Options			Scores
**I have calf or hand cramps ()**	**Yes (3)**	**No (1)**	**Uncertain (2)**	
**After knowing I had osteoporosis, I felt anxious**	**Yes (3)**	**No (1)**	**Uncertain (2)**	
**I do not know much about osteoporosis**	**Yes (3)**	**No (1)**	**Uncertain (2)**	
**I think that osteoporosis is a very serious illness**	**Yes (3)**	**No (1)**	**Uncertain (2)**	
**I have had fractures**	**Yes (3)**	**No (1)**	**Uncertain (2)**	
**I care about the long time period for the treatment of osteoporosis**	**Yes (3)**	**No (1)**	**Uncertain (2)**	
**I am quite worried about fractures**	**Yes (3)**	**No (1)**	**Uncertain (2)**	
**I think osteoporosis can be cured**	**Yes (3)**	**No (1)**	**Uncertain (2)**	
**I feel that treatment of osteoporosis will increase my financial burden**	**Yes (3)**	**No (1)**	**Uncertain (2)**	
**I may keep thinking about my osteoporosis**	**Yes (3)**	**No (1)**	**Uncertain (2)**	
**I may not do much physical exercises because of osteoporosis**	**Yes (3)**	**No (1)**	**Uncertain (2)**	
**I do the housework a little slower, and a little less than before**	**Yes (3)**	**No (1)**	**Uncertain (2)**	
**I may force me to change my eating habits because of osteoporosis**	**Yes (3)**	**No (1)**	**Uncertain (2)**	
**Travel may be restricted because of my osteoporosis**	**Yes (3)**	**No (1)**	**Uncertain (2)**	
**I may have fewer appointments with my relatives, friends, and children**	**Yes (3)**	**No (1)**	**Uncertain (2)**	
**I think osteoporosis can have an impact on my life**	**Yes (3)**	**No (1)**	**Uncertain (2)**	
**My family may take extra care of me because I have osteoporosis**	**Yes (3)**	**No (1)**	**Uncertain (2)**	
**I think I am in poor health**	**Yes (3)**	**No (1)**	**Uncertain (2)**	
**After knowing I have osteoporosis, I feel I am old**	**Yes (3)**	**No (1)**	**Uncertain (2)**	
**It takes a lot of effort to bend my waist to do things**	**Yes (3)**	**No (1)**	**Uncertain (2)**	
**I often feel pains all over the body**	**Yes (3)**	**No (1)**	**Uncertain (2)**	
**I may get upset because of pains**	**Yes (3)**	**No (1)**	**Uncertain (2)**	
**I seem to have a kyphosis**	**Yes (3)**	**No (1)**	**Uncertain (2)**	
**I am aware that I am getting shorter**	**Yes (3)**	**No (1)**	**Uncertain (2)**	
**I am afraid to go out alone or stay home alone.**	**Yes (3)**	**No (1)**	**Uncertain (2)**	
**The pain caused by osteoporosis interferes with my sleep**	**Yes (3)**	**No (1)**	**Uncertain (2)**	
**Sometimes I have a loose tooth or a removed tooth**	**Yes (3)**	**No (1)**	**Uncertain (2)**	
**I have tinnitus or hearing loss**	**Yes (3)**	**No (1)**	**Uncertain (2)**	
**I have memory loss**	**Yes (3)**	**No (1)**	**Uncertain (2)**	
**I have hyperostosis, herniated disks, or spinal deformities**	**Yes (3)**	**No (1)**	**Uncertain (2)**	
**Pre-treatment score**	**Post-treatment score**			

**Note: the scale is answered by the patient without the physician being involved. The total score of 54 points or less is defined as a normal quality of life, > 54–70 points as decreased quality of life, 71–80 points as the quality of life decreases obviously, and > 80 points as a serious decline in quality of life**

### Blood testing

1. Sex hormones. Fasting blood samples will be draw every month before and after treatment by the nurse. Chemiluminescence immunoassay (CLIA) will be used to determine serum sex-hormone levels include estradiol (E2), FSH (Model: Berne, Switzerland, ELECSYS2011).
2. Antioxidant index. Before and after intervention, a 5-ml venous blood sample will be obtained under non-fasting conditions. Samples will be frozen at − 70 °C. Advanced oxidation protein products (AOPP), and total antioxidant capacity (TAC) (with 2,2-diphenyl-1-picrylhydrazyl (DPPH) oxidation) will be measured using the following methods: Walwadkar [18], Kataaha et al. [19], Girbal et al. [20], Kitajima [21] and Janaszewska et al. [22].
3. Bone metabolism index. Fasting venous blood will be collected before treatment, one course of treatment and at the end of treatment, respectively. The enzyme-linked immunosorbent assay (ELISA) (biocell enzyme standard instrument) will be used to determine bone gla protein (BGP) and bone alkaline phosphatase (BALP) before and after treatment
4. Pathway signaling proteins. Western blot will be used to detect the expression of β-catenin, FoxO3a and PPARγ2 protein in bone tissue (Multiskan spectrum, Shanghai, China, ThermoScientific, USA).
5. Follow-up is at 6 months after the completion of the treatment. Follow-up will be performed by telephone to ask if the participants have sustained any new fracture or have experienced recurrence of pain.

### Quality control

In order to ensure the quality of the research, all researchers must attend all training courses and pass the training examinations. They must master all the details of the procedures before they can carry them out. For example, they must master the use of randomization and fill out a case report form (CRF). Acupuncturists should undergo strict and systematic training. The assessor should know how to collect and input data accurately and completely.

To ensure trial quality, clinical monitors designated by the principal investigator will periodically verify all process details.

### Data management

Researchers who have read and understood the standard operating procedures will obtain written consent and collect and manage the study data from the participants, who will be given ongoing encouragement to complete the study. They are telephoned in advanced of each visit and the importance of taking the investigational treatments at the correct times is reinforced. If a participant needs to be withdrawn from the study, the scheduled tests are still performed at the final study visit if the participant is agreeable. If a follow-up investigation is required, researchers will contact the participant by telephone as necessary. After the end of the trial, data entry will be by double entry, and matching will be conducted after inconsistent data has been reviewed. When the data is matched, a data clarification form will be completed and validated.

### Data analysis

The analysis data set will consist of an intention-to-treat data set, a per-protocol (PP) data set, and a safety data set. The intention-to-treat data set will include all subjects assigned to each group. The PP data set will include only participants who adhered to the study protocol and completed the clinical study. The minimum compliance rate for participants taking the acupuncture treatments in the PP data set is 80%. The safety data set will include any participants who were randomly assigned to, and received, at least 1.5 months of the acupuncture treatments. The intention-to-treat analysis will be the primary analysis and will be compared with the PP analysis in a sensitivity analysis. The last-observation-carried-forward method will be used to manage missing data.

All collected data results were compiled using the SPSS 21.0 statistical software package, and collated, checked and statistically analyzed. The comparison between the data groups was performed by using a *χ*^2^ test, and the measurement data was consistent with the normal distribution and the homogeneity of the variance. The mean ± standard deviation ($\overline{\mathrm{x}}\pm \mathrm{S}$) indicates that the rank-sum test is used for comparison between grade data sets that do not satisfy the normal distribution and the homogeneity of the variance. *P* < 0.05 was significant for the difference. The variance analysis will be performed for the difference between the two groups and within the group. We will perform a regression analysis to examine the causal relationship between the E2, TAC and β-catenin, and the primary outcome. The statisticians who are independent of the research team will conduct the data analysis.

### Data monitoring

The Data Monitoring Committee (DMC) of Dali University which is independent from the sponsor and any competing interests will conduct regular monitoring to ensure the quality of the data. Its monitors check that the randomly assigned study participants meet the inclusion and exclusion criteria and that the study is proceeding well, and they ensure that the data is adequately recorded in the case report forms. There will be no interim analysis. The study will continue until the 104 participants have completed the study.

### Adverse events

Safety will be assessed by body temperature, pulse rate and character, respiratory rate, blood pressure, renal function test, and liver function test. These indicators are detected during the period of screening and after 12-week treatment. We will monitor adverse events for each treatment during the trial, including hematomas, skin burns, skin infections and acute pain. Any adverse events or reactions that are thought to be causally associated with the intervention will be recorded, managed, and reported to the study coordinators. Serious adverse reactions will be reported to the Ethical Committee.

## Discussion

Acupuncture therapy is a major component of TCM and is increasingly widely used because it is non-invasive and has fewer side effects [23–25]. Drugs for PMOP exists but have some side effects [8], and their long-term effects are unsatisfactory. Some clinical studies have reported the effect of acupuncture on patients with osteoporosis. A recent meta-analysis reported that the quality of evidence was generally low and most studies had small sample sizes (ranging from 30 to 100); therefore, the evidence for the use of acupuncture in postmenopausal women with osteoporosis is not conclusive, and more high-quality, large-sample, multi-center, and well-designed randomized controlled trials should be conducted to provide a reliable basis for further confirmation of the exact efficacy of acupuncture for PMOP [26]. Compared with previous studies, we combined the TCM therapy with a scientific and rigorous experimental design to explore the effects of OA on women with PMOP. The selection of acupoints and herbs is based on the syndrome differentiation in order to adequately demonstrate the dialectical treatment of TCM.

The evidence for the long-term efficacy of acupuncture in patients with PMOP is not sufficient. This trial will conduct a 6-month follow-up of the patient to observe the long-term efficacy of the OA treatment for PMOP. However, a neurological study [27] showed that although SA and placebo had the same effectiveness as manual acupuncture in terms of reduction of symptoms and objective physiological outcomes, verum acupuncture was superior to SA in improving both peripheral and brain neuro-physiological outcomes.

Among menopausal women, bone mineral density decreases while bone fragility increases, and lumbar spine and femoral fractures are often the common end results for these patients. As ovarian function of postmenopausal women declines, so estrogen deficiency appears. In this process, lipid peroxidation and an oxidative stress state [28] are induced. Oxidative stress can induce aging of BMSCs in the bone marrow, and hinder the differentiation of BMSCs into osteoblasts [29], which leads to osteoporosis. This mechanism includes the involvement of transcription factor FoxO3a, BMSC osteogenic differentiation key factor β-catenin and lipogenic key-factor PPARγ [30]. Oxidative stress induces the binding of core protein β-catenin with FoxO3a and PPARγ in the pathway, which inhibits the osteogenic differentiation of bone marrow which becomes lipogenic differentiation. The oxidative stress induced because of the decreased estrogen level in these patients results in the adipogenic differentiation and imbalance of osteogenic differentiation of BMSCs. Early clinical observation of OA can effectively improve the bone density, reduce pain, improve the quality of daily life of osteoporosis patients, and is the same for PMOP patients. Therefore, the subject uses the OA method, and selects bone density, hormone levels, antioxidant index, bone metabolism index, and pathway index to prove the efficacy, safety and mechanism of OA for PMOP. Our hypothesis is that OA can improve hormone levels which plays an antioxidant role, adjusts the imbalance of adipogenic differentiation and osteogenic differentiation of BMSCs, increases the formation of osteoblasts, increases bone density, reduces the pain experienced by patients, improves the quality of daily life of patients, prevents the occurrence of fractures, and improves the clinical outcome of PMOP patients.

## Trial status

This trial was registered at the Chinese Clinical Trial Registry on 10 August 2018, with ID ChiCTR1800017581. At the time of initial manuscript submission, recruitment had already started (1 August 2018), but it has not been completed. The last patient is expected to be included in the study on 1 August 2020. I confirm that this the protocol, version number 1.0, is the version registered on the date that I have provided.

## Supplementary information

**Additional file 1.** Standard Protocol Items: Recommendations for Interventional Trials (SPIRIT) 2013 Checklist: recommended items to address in a clinical trial protocol and related documents*.

## Data Availability

The data sets during and/or analyzed during the current study available from the corresponding author on reasonable request.

## Abbreviations

PMOP: Postmenopausal osteoporosis
OA: Overall adjustment acupuncture
SA: Sham acupuncture
BMD: Bone mineral density
E2: Estradiol
FSH: Follicle-stimulating hormone
BGP: Bone gla protein
BALP: Bone alkaline phosphatase
TAC: Total antioxidant capacity
DXA: Dual-energy x-ray absorptiometry
AOPP: Advanced oxidation protein products
VAS: Visual analog pain scale score
TCM: Traditional Chinese medicine
QOL: Quality of daily life
BMSCs: Bone-marrow mesenchymal stem cells
SD: Standard deviation
